# DLA Class II Alleles Are Associated with Risk for Canine Symmetrical Lupoid Onychodystropy (SLO)

**DOI:** 10.1371/journal.pone.0012332

**Published:** 2010-08-23

**Authors:** Maria Wilbe, Martine Lund Ziener, Anita Aronsson, Charlotte Harlos, Katarina Sundberg, Elin Norberg, Lisa Andersson, Kerstin Lindblad-Toh, Åke Hedhammar, Göran Andersson, Frode Lingaas

**Affiliations:** 1 Department of Animal Breeding and Genetics, Biomedical Centre, Swedish University of Agricultural Sciences (SLU), Uppsala, Sweden; 2 Fredrikstad Dyrehospital, Gamle Fredrikstad, Norway; 3 Department of Oncology-Pathology, Cancer Centrum Karolinska, Karolinska Institutet, Stockholm, Sweden; 4 Department of Clinical Sciences, Swedish University of Agricultural Sciences (SLU), Uppsala, Sweden; 5 Department of Medical Biochemistry and Microbiology, Uppsala University, Uppsala, Sweden; 6 Broad Institute, Cambridge, Massachusetts, United States of America; 7 Department of Basic Sciences and Aquatic Medicine, Norwegian School of Veterinary Science, Oslo, Norway; Albert Einstein Institute for Research and Education, Brazil

## Abstract

Symmetrical lupoid onychodystrophy (SLO) is an immune-mediated disease in dogs affecting the claws with a suggested autoimmune aethiology. Sequence-based genotyping of the polymorphic exon 2 from DLA-DRB1, -DQA1, and -DQB1 class II loci were performed in a total of 98 SLO Gordon setter cases and 98 healthy controls. A risk haplotype (DRB1*01801/DQA1*00101/DQB1*00802) was present in 53% of cases and 34% of controls and conferred an elevated risk of developing SLO with an odds ratio (OR) of 2.1. When dogs homozygous for the risk haplotype were compared to all dogs not carrying the haplotype the OR was 5.4. However, a stronger protective haplotype (DRB1*02001/DQA1*00401/DQB1*01303, OR = 0.03, 1/OR = 33) was present in 16.8% of controls, but only in a single case (0.5%). The effect of the protective haplotype was clearly stronger than the risk haplotype, since 11.2% of the controls were heterozygous for the risk and protective haplotypes, whereas this combination was absent from cases. When the dogs with the protective haplotype were excluded, an OR of 2.5 was obtained when dogs homozygous for the risk haplotype were compared to those heterozygous for the risk haplotype, suggesting a co-dominant effect of the risk haplotype. In smaller sample sizes of the bearded collie and giant schnauzer breeds we found the same or similar haplotypes, sharing the same DQA1 allele, over-represented among the cases suggesting that the risk is associated primarily with DLA-DQ. We obtained conclusive results that DLA class II is significantly associated with risk of developing SLO in Gordon setters, thus supporting that SLO is an immune-mediated disease. Further studies of SLO in dogs may provide important insight into immune privilege of the nail apparatus and also knowledge about a number of inflammatory disorders of the nail apparatus like lichen planus, psoriasis, alopecia areata and onycholysis.

## Introduction

Canine symmetrical lupoid onychodystrophy (SLO) is described as separation and sloughing of several claws from claw beds and ultimately affecting all claws. The pathogenesis of SLO is incompletely elucidated, but allergic, infectious and immune-mediated diseases have all been associated with symmetrical onychomadesis [1]. Thus, the lupoid reaction observed histopathologically and the clinical signs of onychomadesis represent an immune-mediated disease of the claw, rather than an actual triggering event of the disease. Initially, separation of claw from claw bed and subsequent sloughing is noted on one or more claws, but within two to three months all claws might be affected. Re-growth results in dystrophic claws, brittle, crumbling and misshapen claws [2]. A thorough clinical investigation is important for a correct diagnosis [3]. Histopathology of affected claws present infiltrates of mononuclear cells, apoptosis of the epidermal basal cells and hydropic degeneration of the epidermal basal cells. Typically the inflammatory infiltrate forms a parallel band to the basement membrane called lichenoid pattern. Pigmentary incontinence is also often observed in the dermis [4]. Treatment with immunosuppressive drugs, such as glucocorticoids, has been reported to be successful [5] as well as fatty acid supplementation [6] suggesting an autoimmune aethiology of the disease.

The genomic structure in the dog is unique, with long haplotype blocks and extensive linkage disequilibrium. Dog and human share a similar set of orthologous genes, lives in the same environment and are affected by diseases of similar aethiology. Therefore, the dog is an excellent model for studies on genetic diseases [7]–[9].

Autoimmune diseases in humans have complex patterns of inheritance [10]. In humans, several keratin disorders exist with similarities to SLO [11]. Inflammatory diseases of the nail are common. This includes the group of non-infectious inflammatory disorders of the nail apparatus like psoriasis, lichen planus and other lichenoid diseases, alopecia areata, pemphigus vulgaris, as well as erythema multiforme [12]. Nail changes in lupus erythematosus are not common, but in acute lupus erythematosus, onycholysis is the most common symptom. Nail loss is uncommon but has been observed. Lichen planus is affecting the nail more often than lupus erythematosus and may lead to complete nail loss, either insidiously or relatively rapidly in case of ulcerating lichen planus [12]. In dogs, SLO is a significant health concern and appears to be the most common immune-mediated claw disease [4]. Particularly high disease prevalence has been reported in certain breeds such as Gordon setters (12.6%) [13], [2]. English setters, giant schnauzers (10%) and also bearded collies are reported to have a high incidence of SLO (unpublished observation). The increased risk in specific breeds suggests a significant genetic component, influencing the power of mapping the genetic risk factors underlying SLO. The inheritance of the disease has not been reported but is suggested to be complex. Studies on pedigrees, indicate large differences in incidence between breeding lines and an increased risk in relatives of affected dogs (unpublished observation) supporting a high heritability for the diseases.

In the dog, the major histocompatibility complex (MHC) class II is called DLA (dog leukocyte antigen) class II and consists of three highly polymorphic genes known as DLA-DRB1, DLA-DQA1 and DLA-DQB1 and one monomorphic gene, DLA-DRA [14], [15]. Due to the high linkage disequilibrium in the region and the extensive polymorphism in exon 2 (which encodes the antigen-binding domain), genetic typing of this exon is likely to detect most of the variation in the locus. There are currently 106 DLA-DRB1, 26 DLA-DQA1 and 62 DLA-DQB1 alleles identified in the dog [15]. The DLA class II genes are in high linkage disequilibrium (LD). However, the extension of LD within this region remains unknown. Many previous studies have identified DLA class II as a genetic risk or protective factor for various autoimmune or immune-mediated diseases in dogs [16]-[22]. In humans the association to MHC class II is also well established in autoimmune diseases [16].

The aim of this study was to elucidate the potential associations between DLA class II and risk of developing SLO.

## Results

### Haplotypes, alleles and genotypes

In 196 Gordon setters, a total of 10 different DLA-DRB1/DQA1/DQB1 haplotypes were identified. The majority of the dogs (43.4%) carried haplotype DRB1*01801/DQA1*00101/DQB1*00802. The other nine haplotypes ranged in frequencies between 0.3–12.8% (Table 1). Also, 10 DRB1 alleles, six DQA1 alleles and eight DQB1 alleles were found in the population (Table 2). DRB1*01801 was the most common allele with a frequency of 43.4 % and the other nine DRB1 alleles had frequencies between 0.3–12.8%. 74.2% of Gordon setters had allele DQA1*00101 and the other alleles ranged in frequency between 0.3–11.2%. Eight DQB1 alleles were found with DQB1*00802 being the most common (43.4%) and the others in frequencies between 0.3–25.0%. We also identified 34 different genotypes (haplotype combinations) with frequencies between 0.5–18.9% (Table S1).

**Table 1:** Haplotype frequencies in Gordon setter.

Number	Haplotype DRB1/DQA1/DQB1	Total population % (392)	Cases % (196)	Controls % (196)
1	01801/00101/00802	43.4 (170)	**52.6 (103)**	**34.2 (67)**
2	01501/00601/02301	4.6 (18)	2.6 (5)	6.6 (13)
3	1800103/00101/00201	8.2 (32)	8.2 (16)	8.2 (16)
4	00101/00101/00201	12.8 (50)	15.3 (30)	10.2 (20)
5	02001/00401/01303	8.7 (34)	**0.5 (1)**	**16.8 (33)**
6	04901/01001/01901	11.2 (44)	12.8 (25)	9.7 (19)
7	00901/00101/008011	5.9 (23)	1.5 (3)	10.2 (20)
8	00601/005011/00701	0.3 (1)	0.0 (0)	0.5 (1)
9	00501/00301/00501	1.0 (4)	0.5 (1)	1.5 (3)
10	10102/00101/00201	4.1 (16)	6.1 (12)	2.0 (4)

A total of 10 different haplotypes were found. DRB1*01801/DQA1*00101/DQB1*00802 had an increased frequency in cases and DRB1*02001/DQA1*00401/DQB1*01303 was significantly more frequent in controls, both numbers shown in bold.

**Table 2:** DLA DRB1/DQA1/DQB1 allele frequencies in Gordon setter.

Allele	Total population % (392)	Cases % (196)	Controls % (196)
**DRB1**			
01801	43.4 (170)	**52.6 (103)**	**34.2 (67)**
01501	4.6 (18)	2.6 (5)	6.6 (13)
1800103	8.2 (32)	8.2 (16)	8.2 (16)
00101	12.8 (50)	15.3 (30)	10.2 (20)
02001	8.7 (34)	**0.5 (1)**	**16.8 (33)**
04901	11.2 (44)	12.8 (25)	9.7 (19)
00901	5.9 (23)	1.5 (3)	10.2 (20)
00601	0.3 (1)	0.0 (0)	0.5 (1)
00501	1.0 (4)	0.5 (1)	1.5 (3)
10102	4.1 (16)	6.1 (12)	2.0 (4)
**DQA1**			
00101	74.2 (291)	**83.7 (164)**	**64.8 (127)**
00601	4.6 (18)	2.6 (5)	6.6 (13)
00401	8.7 (34)	**0.5 (1)**	**16.8 (33)**
01001	11.2 (44)	12.8 (25)	9.7 (19)
005011	0.3 (1)	0.0 (0)	0.5 (1)
00301	1.0 (4)	0.5 (1)	1.5 (3)
**DQB1**			
00802	43.4 (170)	**52.6 (103)**	**34.2 (67)**
02301	4.6 (18)	2.6 (5)	6.6 (13)
00201	25.0 (98)	29.6 (58)	20.4 (40)
01303	8.7 (34)	**0.5 (1)**	**16.8 (33)**
01901	11.2 (44)	12.8 (25)	9.7 (19)
008011	5.9 (23)	1.5 (3)	10.2 (20)
00701	0.3 (1)	0.0 (0)	0.5 (1)
00501	1.0 (4)	0.5 (1)	1.5 (3)

Altogether, 10 DRB1 alleles, six DQA1 alleles and eight DQB1 alleles were found in the population. The alleles DRB1*01801, DQA1*00101 and DQB1*00802 was observed in a higher frequency in cases while the alleles DRB1*02001, DQA1*00401and DQB1*01303 was more frequent in controls. Numbers in bold indicate significant different between cases and controls.

Five haplotypes were found in the 10 bearded collies. The two most common, DRB1*01801/DQA1*00101/DQB1*00802 and DRB1*01801/DQA1*00101/DQB1*00201 had frequencies of 50% and 30%, respectively. The other haplotypes ranged in frequencies between 5–10% (Table S2). There were three DRB1 alleles (*01801, *00201 and *01501) and three DQA1 alleles (*00101, *00901 and *00601). Five DQB1 alleles were identified. Two were common (30–50%) and three were rare (5–10%) (Table S3). A total of four different genotypes were found (Table S1).

In the 110 giant schnauzers, we identified 10 haplotypes. Four were common (13.6–23.6%) and six were rare (0.5–9.%) (Table S4). We also identified eight DRB1 alleles, four DQA1 alleles and six DQB1 alleles (Table S5). We found 32 genotypes (frequencies of 0.9–10.0%) (Table S1).

### Disease association

In Gordon setters, two haplotypes differed markedly in frequency between cases and controls. The haplotype DRB1*01801/DQA1*00101/DQB1*00802 occurred in 52.6% of the cases compared to 34.2% of the controls (OR = 2.1, p<0.001) and was defined as a risk-haplotype. This haplotype was also the most common haplotype in the Gordon setter population (43.4%). The allele frequencies of each of the genes involved in the risk haplotype were higher in cases compared to controls and showed a significant disease association (DRB1*01801: 52.6% vs. 34.2%, OR = 2.1, p<0.001; DQA1*00101: 83.7% vs. 64.8%, OR = 2.8, p≤0.001 and DQB1*00802: 52.6% vs. 34.2% OR = 2.1, p≤0.001). All of these alleles are the most common alleles in the population with frequencies of 43.4%, 74.2% and 43.3% (DRB1/DQA1/DQB1, respectively).

A lower frequency of SLO was found in Gordon setter dogs carrying haplotype DRB1*02001/DQA1*00401/DQB1*01303. This haplotype is rare in the population (8.7%) and was found in 16.8% of the controls compared to 0.5% of the cases (OR = 0.03, p≤0.0001) and thus is significantly associated with protection against disease development. Calculation of the allele frequencies at each of the three loci separately showed the same results, as these alleles were unique to this haplotype.

Gordon setter dogs homozygous for the risk haplotype, DRB1*01801/DQA1*00101/DQB1*00802 had even higher odds for developing disease. In fact, 18.9% in the population was homozygous for this risk haplotype, which was found in 28.6% of cases compared to 9.2% of controls (OR = 4.0, p = 0.001 None of the 11 dogs heterozygous for the risk and the protective haplotypes had SLO, emphasizing the strength of the protective allele (Table 3).

**Table 3:** Haplotypes, alleles and genotypes in Gordon setter cases and controls.

Haplotype, allele or genotype	Total population % (392)	Cases % (196)	Controls % (196)	OR	P-value	99% CI
01801/00101/00802	43.4 (170)	52.6 (103)	34.2 (67)	2.1	0.0004	1.3–3.6
02001/00401/01303	8.7 (34)	0.5 (1)	16.8 (33)	0.03	0.0001	0.002–0.35
DRB1*01801	43.4 (170)	52.6 (103)	34.2 (67)	2.1	0.0004	1.25–3.64
DRB1*02001	8.7 (34)	0.5 (1)	16.8 (33)	0.03	<.0001	0.002–0.35
DQA1*00101	74.2 (291)	83.7 (164)	64.8 (127)	2.8	<.0001	1.48–5.23
DQA1*00401	8.7 (34)	0.5 (1)	16.8 (33)	0.03	<.0001	0.002–0.35
DQB1*00802	43.4 (170)	52.6 (103)	34.2 (67)	2.1	0.0004	1.25–3.64
DQB1*01303	8.7 (34)	0.5 (1)	16.8 (33)	0.03	<.0001	0.002–0.35
Genotype 1	18.9 (37)	28.6 (28)	9.2 (9)	3.96	0.001	1.36–11.52
Genotype 5	5.6 (11)	0 (0)	11.2 (11)	0	0.002	0

Odds ratio (OR) and Yates p-values were calculated for differences greater than 10% between cases and controls. A 99% confidence interval (CI) was used.

We also compared the dogs homozygous for risk haplotype vs. those not carrying this haplotype (OR = 5.41, p = 0.0004) and vs. heterozygous for risk haplotype (OR = 3.24, p = 0.01) and finally heterozygous for risk haplotype vs. not carrying risk haplotype (OR = 1.67, p = 0.17) (Table 4). However, when the protective haplotype was removed from the analysis, the odds ratio for homozygosity for the risk haplotype vs. all without risk alleles was 2.97 (Table 4), for homozygotes vs. heterozygotes the OR was 2.52 and for heterozygotes vs. no risk the OR was 1.18 although none of these results were significant based on the smaller sample size.

**Table 4:** Comparison of OR for SLO depending on the number of risk haplotypes.

Haplotype	Cases (n)	Controls (n)	OR	Yates P-value	99% CI
Homozygous risk	28	9	5.41	0.0003	1.64–17.88
No risk	23	40			
Homozygous risk	28	9	2.97	0.04	0.84–10.49
No risk - no protection	22	21			
Homozygous risk	28	9	3.24	0.01	1.06–9.93
Heterozygous risk	47	49			
Homozygous risk	28	9	2.52	0.05	0.81–7.83
Heterozygous risk - no protection	47	38			
Heterozygous risk	47	49	1.67	0.17	0.71–3.92
No risk	23	40			
Heterozygous risk - no protection	47	38	1.18	0.79	0.045–3.10
No risk - no protection	22	21			
Homozygous risk	28	9	93.3	<. 0001	5.68–1532
Protection	1	30			

The difference in OR according to the number of risk haplotypes (homozygous- heterozygous-none). Risk haplotype is DRB1*01801/DQA1*00101/DQB1*00802 and protective haplotype is DRB1*02001/DQA1*00401/DQB1*01303.

### Comparative analysis between breeds

Bearded collies and giant schnauzers were analyzed for comparative purposes, despite small sample numbers. Interestingly, the risk haplotype found in Gordon setter (DRB1*01801/DQA1*00101/DQB1*00802) was also found in increased frequency among the bearded collie cases (cases 40% vs. controls 20%). A similar haplotype (DRB1*01801/DQA1*00101/DQB1*00202), where only the DQB1 allele differs from the risk haplotype was also identified (cases 60% vs. controls 40%). These two haplotypes are the only ones occurring in cases, whereas in controls, three more haplotypes exists. When evaluating the alleles it appears that all bearded collie cases carry DRB1*01801 and DQB1*00101, further supporting the findings in Gordon setter.

In giant schnauzer we observed a higher number of possible cases carrying haplotype DRB1*00101/DQA1*00101/DQB1*00201 compared to controls (26.9% vs. 15.0%). This risk haplotype of DRB1 and DQB1 differs from Gordon setter and bearded collie, but has the same risk DQA1 allele as the risk haplotype found in Gordon setter and bearded collie further supporting the findings in these breeds. When DQA1*00101 was analyzed separately in giant schnauzer it was not significantly associated with an increased risk. We also identified a protective haplotype, DRB1*01301/DQA1*00301/DQB1*00501 in giant schnauzer.

## Discussion

In the present study we have evaluated the association of MHC class II haplotypes and alleles with symmetrical lupoid onychodystrophy in a large cohort of Gordon setter and smaller numbers from two additional breeds of dogs, the giant schnauzer and the bearded collie. The analysis revealed that DLA class II is significantly associated with SLO and provides support for the hypothesis of SLO being an immune-mediated disease. Genetic association between DLA and SLO and similar claw conditions has been established in all the breeds analyzed here even if the number of dogs studied and the level of significance varied between breeds. We found that dogs homozygous for the risk haplotype had elevated risk of 2.97 of developing SLO, but that a rare protective haplotype had a stronger 33-fold protective effect. The protective haplotype had an uneven distribution between cases and controls and only three (10%) of the controls were homozygous. Because 90% of the controls were heterozygotes, including 11 (37%) heterozygotes for the combination of risk and protective haplotypes, the results indicate a dominant effect of the protective haplotype. It is worth emphasizing the very high OR of 93.3 when comparing dogs homozygous for the risk haplotype vs. dogs with at least one protective haplotype, even if the observed number of the protective haplotype is low in cases (Table 4).

The relatively high frequency of dogs homozygous for DLA class II haplotypes, provides a unique opportunity to estimate an unbiased risk effect of the most common haplotypes, and to compare homozygote vs. heterozygote dogs. In humans, homozygosity for HLA haplotypes is extremely rare, preventing analyses of genetic risk association between MHC class II and immune-mediated disease in homozygotes. The comparison of OR depending on the presence of two (homozygous) or one (heterozygous) risk haplotype should be carefully interpreted (Table 4). Comparison of the group of dogs being homozygous for the risk haplotype to those with no risk haplotype (OR = 5.41), to the heterozygotes (OR = 3.24) and the heterozygotes versus those with no risk (OR = 1.67) could indicate a co-dominant effect of the risk haplotype.

When we compared the SLO risk haplotype in the Gordon setter, with those of the bearded collie and the giant schnauzer, we observed that the DRB1-DQA1-DQB1 haplotypes were relatively similar although the sample number was small. Even if there were some differences, all the major risk haplotypes in the three breeds contained the same DQA1 allele. Strikingly, the DQA1*00101 molecule associated with SLO has an Arg at position 55 instead of an Asp as well as (DQA1*00601, *00901, *01001, *01301, *01401 and *01501). Kennedy et al suggested that this amino acid is likely to be important for peptide binding to the canine DQ class II molecule [23], but this remains to be verified. One might speculate that some antigenic peptides bound by the Arg containing DQ molecules leads to increased risk of aberrant immune responses ultimately leading to de-regulated immunity and disease. The exact type of immune-mediated mechanism involved in development of SLO remains to be established. In several human autoimmune diseases, MHC class II-mediated autoantigen presentation leads to broken tolerance and similar mechanisms are likely to operate during SLO progression.

MHC class II-mediated autoantigen presentation leading to broken tolerance is likely to operate during SLO progression. The autoantigen/s involved in SLO remains to be defined. Identification of such antigens could lead to increased understanding of similar diseases in human.

The human hair follicles and the nail apparatus is reported to harbour an unusual site of immune reactions called immune privilege [24], originally described as sites providing a relative protection against rejection of transplants [25]. There are strong indications that parts of the hair follicle and nail epithelium is characterized by an absence of or very low expression of some MHC antigens. These mechanisms remain to be fully understood, but there are reasons to believe that a disturbance of these mechanisms may play a key role in the pathogenesis of one of the most common organ-specific autoimmune diseases, alopecia areata, which shows frequent nail involvement [12], [24], [26].

In summary, we have identified both a risk haplotype for developing SLO in Gordon setter and also a haplotype that may protect dogs from developing SLO. The risk is even higher in dogs homozygous for the specific risk haplotype. In contrast, dogs heterozygous for the risk and protective haplotypes are over-represented among controls suggesting that the protective effect is phenotypically stronger. To be able to distinguish a potential functional effect of DRB1*1801 from the closely linked loci in strong LD, it will be important to type more dogs/breeds to identify dogs with DRB1*1801. However, other variants of linked loci, but our findings may provide an impetus for future breeding practices in an effort to increase the frequency of the protective haplotype to reduce the incidence of SLO in Gordon setters. Moreover, canine SLO could provide new information that could benefit a number of related non-infectious inflammatory disorders of the nail apparatus in humans including lichen planus, psoriasis, alopecia areata, pemphigus vulgaris, and onycholysis and provide new insight in immune privilege of the nail apparatus.

## Materials and Methods

### Study population

The main study population was based on Gordon setters. A total of 196 dogs were included, among these, 98 dogs were classified as SLO cases and 98 as healthy controls. Most of the cases were unrelated at the grand-parental level. The average age of cases were 5.6 years of age while the control dogs had an average age of 10 years (Table S6).

We also used 10 bearded collies and 110 giant schnauzers for comparative purpose. Five bearded collies were defined as SLO cases and five as healthy controls. In giant schnauzer, we used 30 healthy controls and 80 dogs classified with different claw abnormalities. A total of seven SLO cases were identified among the giant schnauzer population. The age of onset for SLO in both giant schnauzers and Gordon setters is between two and seven years of age, with an average of about 4.5 years of age. All the control dogs used for comparative purpose in this study were older than seven years of age and unrelated (Table S6).

### Clinical examination

The inclusion criteria used for cases were veterinary-verified diagnosis, where dogs two to seven years of age lost all claws on all four paws within a short time span. Inclusion criteria for all the control dogs were that they had never experienced claw disorders and were more than eight years of age for the Gordon setters and more than seven years of age for all the other breeds. The giant schnauzers were classified according to owner's questionnaire and grouped according to Table S7.

### Isolation of genomic DNA

Genomic DNA from the Gordon setters was extracted using 250 µl EDTA blood using the E.Z.N.A. Blood DNA Kit (Omega bio-tek, Norcross, GA).

Genomic DNA from bearded collies and giant schnauzers was extracted from 200 µl EDTA blood by using a standard salt extraction protocol, the Qiagen QIAamp DNA Blood Mini Kit (Qiagen, Valencia, CA).

### PCR amplification, DNA Sequencing and data analysis

The methods for amplification, sequencing and data analysis of DLA-DRB1, -DQA1 and -DQB1 were performed as previously described [21]. Primer pairs used for amplification of DLA-DRB1, -DQA1, and -DQB1 was DRB1 (forward): gatccccccgtccccacag, DRB1 (reverse): cgcccgctgcgctca, DQA1 (forward): taaggttcttttctccctct, DQA1 (reverse): ggacagattcagtgaagaga, DQB1 (forward): ctcactggcccggctgtctc and DQB1 (reverse): cacctcgccgctgcaacgtg [14]. A T7 tail (taatacgactcactatag) was used to label the PCR products for sequencing. The purified PCR products were sequenced using capillary-electrophoresis on an Applied Biosystems 3730. BigDye® Terminator v3.1 (Applied Biosystems, Foster City, CA). Finally, the nucleotide sequences for DLA-DRB1, -DQA1, and -DQB1 were analyzed using MatchTools and MatchToolsNavigator (Applied Biosystems) also used for assigning alleles, haplotypes and genotypes. The most frequently occurring haplotypes could easily be identified in homozygous dogs. Due to the high frequency of these haplotypes, the probability that they are present also in heterozygous dogs is high. After thorough manual checking of each diploid sequence, additional haplotypes not found in homozygous individuals could be identified by “subtracting” the haplotypes initially identified in homozygotes. All alleles identified in this study are available in the IPD - MHC Database (http://www.ebi.ac.uk/ipd/mhc/dla/index.html).

### Statistical analyses

Statistical analyses were performed with the statistical program VassarStats (http://faculty.vassar.edu/lowry/VassarStats.html). 2×2 Contingency tables were used for calculations of Odds ratio and Yates p-value, which is corrected for continuity. The number of SLO-cases and controls having each specific allele, haplotype or genotype compared to the overall number of cases and controls in dogs not carrying it, was the basis for the OR-estimates. We also evaluated dogs homozygous for the risk haplotype vs. dogs heterozygous for the risk haplotype, dogs homozygous for the risk haplotype vs. the other haplotypes and dogs heterozygous for the risk haplotype vs. all other haplotypes. A 99% confidence interval (CI) was used for all tests. Statistical analysis was only performed for Gordon setters, due to an insufficient number of dogs in bearded collies and uncertain disease classification in giant schnauzers.

### Ethics Statement

Ethical approval for performing this study has been granted by the Ethical board for experimental animals in Uppsala, Sweden (Dnr C138/6).

## Supporting Information

Table S1 Genotype frequencies in Gordon setter, bearded collie and giant schnauzer. Genotype 1 in Gordon setter (being homozygous for DRB1*01801/DQA1*00101/DQB1*00802) gave an even higher risk for developing SLO and genotype 5 (DRB1*01801/DQA1*00101/DQB1*00802 and DRB1*02001/DQA1*00401/DQB1*01303) was protective.
(0.10 MB DOC)

Table S2 Haplotype frequencies in bearded collie. Only two different haplotypes were found in cases whereas five were found in control dogs. Haplotype 1 and 2 occur in higher frequency in cases compared to controls (not significant).
(0.03 MB DOC)

Table S3 DLA DRB1/DQA1/DQB1 allele frequencies in bearded collies. Altogether, three DRB1, three DQA1 and five DQB1 alleles were found in the population. Only one DRB1, one DQA1 and two DQB1 alleles were identified in the cases compared to three DRB1, three DQA1 and five DQB1 alleles in control dogs.
(0.03 MB DOC)

Table S4 Haplotype frequencies in giant schnauzer. 10 different haplotypes were identified in the total population. Haplotype DRB1*00101/DQA1*00101/DQB1*00201, was more common in cases compared to control dogs. Haplotype DRB1*01301/DQA1*00301/DQB1*00501 was more frequently occurring in controls compared to cases.
(0.04 MB DOC)

Table S5 DLA DRB1/DQA1/DQB1 allele frequencies in giant schnauzer. Altogether, eight DRB1 alleles, four DQA1 alleles and six DQB1 alleles were found in the population. The allele DRB1*00101 was increased in cases compared to controls. A protective effect was found for dogs carrying allele DRB1*01301.
(0.04 MB DOC)

Table S6 Diagnostic information and DLA-DRB1, -DQA1 and -DQB1 alleles for all dogs included in the study (Gordon Setter (GSet), bearded collie (BC) and giant schnauzer (GSch)).
(0.51 MB DOC)

Table S7 The inclusion criteria used for giant schnauzers.
(0.03 MB DOC)
