# Correction: DLA Class II Alleles Are Associated with Risk for Canine Symmetrical Lupoid Onychodystrophy (SLO)

**DOI:** 10.1371/annotation/6f88cccd-2f41-464d-904b-24356b5d87c9

**Published:** 2010-09-23

**Authors:** Maria Wilbe, Martine Lund Ziener, Anita Aronsson, Charlotte Harlos, Katarina Sundberg, Elin Norberg, Lisa Andersson, Kerstin Lindblad-Toh, Åke Hedhammar, Göran Andersson, Frode Lingaas

The last word in the title is misspelled. The correct spelling is "onychodystrophy." The title should therefore read: DLA Class II Alleles Are Associated with Risk for Canine Symmetrical Lupoid Onychodystrophy (SLO). The corrected citation is: Wilbe M, Ziener ML, Aronsson A, Harlos C, Sundberg K, et al. (2010) DLA Class II Alleles Are Associated with Risk for Canine Symmetrical Lupoid Onychodystrophy (SLO). PLoS ONE 5(8): e12332. doi:10.1371/journal.pone.0012332

