# Supplementary material for: DLA Class II Alleles Are Associated with Risk for Canine Symmetrical Lupoid Onychodystropy (SLO)
Source: PLoS One. 2010 Aug 23;5(8):e12332. doi: 10.1371/journal.pone.0012332 (PMC2925901; doi:10.1371/journal.pone.0012332)
Supplement: Table S1 — Genotype frequencies in Gordon setter, bearded collie and giant schnauzer. Genotype 1 in Gordon setter (being homozygous for DRB1*01801/DQA1*00101/DQB1*00802) gave an even higher risk for developing SLO and genotype 5 (DRB1*01801/DQA1*00101/DQB1*00802 and DRB1*02001/DQA1*00401/DQB1*01303) was protective. (0.10 MB DOC) [file pone.0012332.s001.doc]

| **Haplotype** | **Genotype** | **Total population %** | **Cases %** | **Controls %** |
| --- | --- | --- | --- | --- |
| **Gordon setter** |  | % (196) | %(98) | % (98) |
| 1, 1 | 1 | 18.9 (37) | **28.6 (28)** | **9.2 (9)** |
| 1, 2 | 2 | 4.1 (8) | 2.0 (2) | 6.1 (6) |
| 1, 3 | 3 | 9.7 (19) | 14.3 (14) | 5.1 (5) |
| 1, 4 | 4 | 9.7 (19) | 12.1 (12) | 7.1 (7) |
| 1, 5 | 5 | 5.6 (11) | **0 (0)** | **11.2 (11)** |
| 1, 6 | 6 | 5.1 (10) | 6.1 (6) | 4.1 (4) |
| 1, 7 | 7 | 6.6 (13) | 2.0 (0) | 11.2 (11) |
| 1, 8 | 8 | 0.5 (1) | 0 (0) | 1.0 (1) |
| 1, 9 | 9 | 1.0 (2) | 1.0 (1) | 1.0 (1) |
| 1, 10 | 10 | 6.6 (13) | 10.2 (10) | 3.1 (3) |
| 2, 2 | 11 | 0.5 (1) | 1.0 (1) | 0 (0) |
| 2, 3 | 12 | 1.0 (2) | 0 (0) | 2.0 (2) |
| 2, 5 | 13 | 2.0 (4) | 0 (0) | 4.1 (4) |
| 2, 6 | 14 | 0.5 (1) | 0 (0) | 1.0 (1) |
| 2, 7 | 15 | 0.5 (1) | 1.0 (1) | 0 (0) |
| 3, 3 | 16 | 0.5 (1) | 0 (0) | 1.0 (1) |
| 3, 4 | 17 | 1.5 (3) | 1.0 (1) | 2.0 (2) |
| 3, 5 | 18 | 1.5 (3) | 0 (0) | 3.1 (3) |
| 3, 6 | 19 | 1.0 (2) | 1.0 (1) | 1.0 (1) |
| 3, 7 | 20 | 0.5 (1) | 0 (0) | 1.0 (1) |
| 4, 4 | 21 | 2.0 (4) | 4.1 (4) | 0 (0) |
| 4, 5 | 22 | 3.6 (7) | 1.0 (1) | 6.1 (6) |
| 4, 6 | 23 | 5.1 (10) | 7.1 (7) | 3.1 (3) |
| 4, 7 | 24 | 1.0 (2) | 0 (0) | 2.0 (2) |
| 4, 10 | 25 | 0.5 (1) | 1.0 (1) | 0 (0) |
| 5, 5 | 26 | 1.5 (3) | 0 (0) | 3.1 (3) |
| 5, 6 | 27 | 0.5 (1) | 0 (0) | 1.0 (1) |
| 5, 7 | 28 | 0.5 (1) | 0 (0) | 1.0 (1) |
| 5, 10 | 29 | 0.5 (1) | 0 (0) | 1.0 (1) |
| 6, 6 | 30 | 4.1 (8) | 5.1 (5) | 3.1 (3) |
| 6, 7 | 31 | 0.5 (1) | 0 (0) | 1.0 (1) |
| 6, 9 | 32 | 1.0 (2) | 0 (0) | 2.0 (2) |
| 6,10 | 33 | 0.5 (1) | 1.0 (1) | 0 (0) |
| 7, 7 | 34 | 1.0 (2) | 0 (0) | 2.0 (2) |
|  |  |  |  |  |
| **bearded collie** |  | % (10) | %(5) | % (5) |
| 1, 2 | 1 | 60 (6) | 80 (4) | 40 (2) |
| 2, 2 | 2 | 20 (2) | 20 (1) | 20 (1) |
| 3, 5 | 3 | 10 (1) | 0 (0) | 20 (1) |
| 4, 5 | 4 | 10 (1) | 0 (0) | 20 (1) |
|  |  |  |  |  |
| **giant schnauzer** |  | % (110) | % (80) | % (30) |
| 1, 1 | 1 | 10.0 (11) | 11.3 (9) | 6.7 (2) |
| 1, 2 | 2 | 7.3 (8) | 6.3 (5) | 10.0 (3) |
| 1, 3 | 3 | 7.3 (8) | 8.8 (7) | 3.3 (1) |
| 1, 4 | 4 | 2.7 (3) | 3.8 (3) | 0 (0) |
| 1, 5 | 5 | 5.5 (6) | 6.3 (5) | 3.3 (1) |
| 1,6 | 6 | 3.6 (4) | 5.0 (4) | 0 (0) |
| 1, 10 | 7 | 0.9 (1) | 1.3 (1) | 0 (0) |
| 2, 2 | 8 | 3.6 (4) | 3.8 (3) | 3.3 (1) |
| 2, 6 | 9 | 5.5 (6) | 6.3 (5) | 3.3 (1) |
| 3, 2 | 10 | 1.8 (2) | 2.5 (2) | 0 (0) |
| 3, 3 | 11 | 5.5 (6) | 7.5 (6) | 0 (0) |
| 3, 4 | 12 | 4.5 (5) | 0 (0) | 16.7 (5) |
| 3, 5 | 13 | 10.0 (11) | 7.5 (6) | 16.7 (5) |
| 3, 6 | 14 | 3.6 (4) | 5.0 (4) | 0 (0) |
| 3, 7 | 15 | 1.8 (2) | 1.3 (1) | 3.3 (1) |
| 3, 8 | 16 | 0.9 (1) | 1.3 (1) | 0 (0) |
| 3, 9 | 17 | 0.9 (1) | 1.3 (1) | 0 (0) |
| 4, 2 | 18 | 0.9 (1) | 1.3 (1) | 0 (0) |
| 4, 4 | 19 | 1.8 (2) | 1.3 (1) | 3.3 (1) |
| 4, 6 | 20 | 1.8 (2) | 2.5 (2) | 0 (0) |
| 5, 2 | 21 | 4.5 (5) | 5.0 (4) | 3.3 (1) |
| 5, 4 | 22 | 1.8 (2) | 0 (0) | 6.7 (2) |
| 5, 5 | 23 | 1.8 (2) | 0 (0) | 6.7 (2) |
| 5, 6 | 24 | 1.8 (2) | 2.5 (2) | 0 (0) |
| 5, 8 | 25 | 4.5 (5) | 5.0 (4) | 3.3 (1) |
| 5, 10 | 26 | 0.9 (1) | 0 (0) | 3.3 (1) |
| 6, 8 | 27 | 0.9 (1) | 1.3 (1) | 0 (0) |
| 6,6 | 28 | 0.9 (1) | 0 (0) | 3.3 (1) |
| 7, 7 | 29 | 0.9 (1) | 0 (0) | 3.3 (1) |
| 10, 8 | 30 | 0.9 (1) | 1.3 (1) | 0 (0) |
| 10, 8 | 31 | 0.9 (1) | 1.3 (1) | 0 (0) |
