# Supplementary material for: DLA Class II Alleles Are Associated with Risk for Canine Symmetrical Lupoid Onychodystropy (SLO)
Source: PLoS One. 2010 Aug 23;5(8):e12332. doi: 10.1371/journal.pone.0012332 (PMC2925901; doi:10.1371/journal.pone.0012332)
Supplement: Table S2 — Haplotype frequencies in bearded collie. Only two different haplotypes were found in cases whereas five were found in control dogs. Haplotype 1 and 2 occur in higher frequency in cases compared to controls (not significant). (0.03 MB DOC) [file pone.0012332.s002.doc]

| **NumberN** | **Haplotype**  **DRB1/DQA1/DQB1** | **Total population**  **% (20)** | **Cases**  **% (10)** | **Controls**  **% (10)** |
| --- | --- | --- | --- | --- |
| 1 | 01801/00101/00802 | 30% (6) | 40% (4) | 20% (2) |
| 2 | 01801/00101/00201 | 50% (10) | 60% (6) | 40% (4) |
| 3 | 01501/00601/00301 | 5% (1) | 0 | 10% (1) |
| 4 | 00201/00901/00101 | 5% (1) | 0 | 10% (1) |
| 5 | 01501/00601/02301 | 10% (2) | 0 | 20% (2) |
