# Supplementary material for: DLA Class II Alleles Are Associated with Risk for Canine Symmetrical Lupoid Onychodystropy (SLO)
Source: PLoS One. 2010 Aug 23;5(8):e12332. doi: 10.1371/journal.pone.0012332 (PMC2925901; doi:10.1371/journal.pone.0012332)
Supplement: Table S3 — DLA DRB1/DQA1/DQB1 allele frequencies in bearded collies. Altogether, three DRB1, three DQA1 and five DQB1 alleles were found in the population. Only one DRB1, one DQA1 and two DQB1 alleles were identified in the cases compared to three DRB1, three DQA1 and five DQB1 alleles in control dogs. (0.03 MB DOC) [file pone.0012332.s003.doc]

| **Allele** | **Total population**  **% (20)** | **Cases**  **% (10)** | **Controls**  **% (10)** |
| --- | --- | --- | --- |
| **DRB1** |  |  |  |
| 01801 | 80 (16) | 100 (10) | 60 (6) |
| 00201 | 5 (1) | 0 | 10 (1) |
| 01501 | 15 (3) | 0 | 30 (3) |
| **DQA1** |  |  |  |
| 00101 | 80 (16) | 100 (10) | 60 (6) |
| 00901 | 5 (1) | 0 | 10 (1) |
| 00601 | 15 (3) | 0 | 30 (3) |
| **DQB1** |  |  |  |
| 00201 | 50 (10) | 60 (6) | 40 (4) |
| 00802 | 30 (6) | 40 (4) | 20 (2) |
| 02301 | 10 (2) | 0 | 20 (2) |
| 00301 | 5 (1) | 0 | 10 (1) |
| 00101 | 5 (1) | 0 | 10 (1) |
