# Supplementary material for: DLA Class II Alleles Are Associated with Risk for Canine Symmetrical Lupoid Onychodystropy (SLO)
Source: PLoS One. 2010 Aug 23;5(8):e12332. doi: 10.1371/journal.pone.0012332 (PMC2925901; doi:10.1371/journal.pone.0012332)
Supplement: Table S4 — Haplotype frequencies in giant schnauzer. 10 different haplotypes were identified in the total population. Haplotype DRB1*00101/DQA1*00101/DQB1*00201, was more common in cases compared to control dogs. Haplotype DRB1*01301/DQA1*00301/DQB1*00501 was more frequently occurring in controls compared to cases. (0.04 MB DOC) [file pone.0012332.s004.doc]

| **Number**  **N** | **Haplotype**  **DRB1/DQA1/DQB1** | **Total population**  **% (220)** | **Cases**  **% (160)** | **Controls**  **% (60)** |
| --- | --- | --- | --- | --- |
| 1 | 00101/00101/00201 | 23,6% (52) | 26,9% (43) | 15% (9) |
| 2 | 01201/00101/00201 | 13,6% (30) | 14,4% (23) | 11,7% (7) |
| 3 | 00601/00401/01303 | 20, 9% (46) | 21,3% (34) | 20% (12) |
| 4 | 01301/00101/00201 | 7,7% (17) | 5,0 % (8) | 15% (9) |
| 5 | 01301/00301/00501 | 16,4% (36) | 13,1% (21) | 25% (15) |
| 6 | 02301/00301/00501 | 9,6% (21) | 11,3% (18) | 5% (3) |
| 7 | 00901/00101/008011 | 1,8 % (4) | 0,6% (1) | 5% (3) |
| 8 | 01501/00601/02201 | 4,1% (9) | 5,0% (8) | 1,7% (1) |
| 9 | 02001/00401/01303 | 0,5% (1) | 0,6% (1) | 0 |
| 10 | 01501/00601/00301 | 1,8% (4) | 1,9% (3) | 1,7% (1) |
