# Supplementary material for: DLA Class II Alleles Are Associated with Risk for Canine Symmetrical Lupoid Onychodystropy (SLO)
Source: PLoS One. 2010 Aug 23;5(8):e12332. doi: 10.1371/journal.pone.0012332 (PMC2925901; doi:10.1371/journal.pone.0012332)
Supplement: Table S5 — DLA DRB1/DQA1/DQB1 allele frequencies in giant schnauzer. Altogether, eight DRB1 alleles, four DQA1 alleles and six DQB1 alleles were found in the population. The allele DRB1*00101 was increased in cases compared to controls. A protective effect was found for dogs carrying allele DRB1*01301. (0.04 MB DOC) [file pone.0012332.s005.doc]

| **Allele** | **Total population**  **% (220)** | **Cases**  **% (160)** | **Controls**  **% (60)** |
| --- | --- | --- | --- |
| **DRB1** |  |  |  |
| 00101 | 23,6 (52) | 26,9 (43) | 15 (9) |
| 00601 | 20,9 (46) | 21,3 (34) | 20 (12) |
| 01301 | 24,1 (53) | 18,1 (29) | 40 (24) |
| 00901 | 1,8 (4) | 0,6 (1) | 5 (3) |
| 01201 | 13,6 (30) | 14,4 (23) | 11,7 (7) |
| 02301 | 9,54(21) | 11,3 (18) | 5 (3) |
| 01501 | 5,9 (13) | 6,9 (11) | 3,3 (2) |
| 02001 | 0,5 (1) | 0,6 (1) | 0 |
| **DQA1** |  |  |  |
| 00101 | 46,8 (103) | 46,9 (75) | 46,7 (28) |
| 00401 | 21,4 (47) | 21,9 (35) | 20 (12) |
| 00301 | 25,9 (57) | 24,4 (39) | 30 (18) |
| 00601 | 5,9 (13) | 6,87 (11) | 3,3 (2) |
| **DQB1** |  |  |  |
| 00201 | 45 (99) | 46,3 (74) | 41,7 (25) |
| 01303 | 21,4 (47) | 21,9 (35) | 20 (12) |
| 00501 | 25,9 (57) | 24,4 (39) | 30 (18) |
| 08011 | 1,8 (4) | 0,6 (1) | 5 (3) |
| 00301 | 1,8 (4) | 1,9 (3) | 1,7 (1) |
| 02201 | 4,1 (9) | 0 | 1,7 (1) |
