# Supplementary material for: DLA Class II Alleles Are Associated with Risk for Canine Symmetrical Lupoid Onychodystropy (SLO)
Source: PLoS One. 2010 Aug 23;5(8):e12332. doi: 10.1371/journal.pone.0012332 (PMC2925901; doi:10.1371/journal.pone.0012332)
Supplement: Table S6 — Diagnostic information and DLA-DRB1, -DQA1 and -DQB1 alleles for all dogs included in the study (Gordon Setter (GSet), bearded collie (BC) and giant schnauzer (GSch)). (0.51 MB DOC) [file pone.0012332.s006.doc]

| **ID** | **Breed** | **Status** | **DRB1** | **DQA1** | **DQB1** | **DRB1** | **DQA1** | **DQB1** | **Haplotype** | **Genotype** |
| --- | --- | --- | --- | --- | --- | --- | --- | --- | --- | --- |
| 1 | GSet | 0 | 01801 | 00101 | 00802 | 01801 | 00101 | 00802 | 1, 1 | 1 |
| 2 | GSet | 0 | 01801 | 00101 | 00802 | 01801 | 00101 | 00802 | 1, 1 | 1 |
| 3 | GSet | 0 | 01801 | 00101 | 00802 | 01801 | 00101 | 00802 | 1, 1 | 1 |
| 4 | GSet | 0 | 01801 | 00101 | 00802 | 01801 | 00101 | 00802 | 1, 1 | 1 |
| 5 | GSet | 0 | 01801 | 00101 | 00802 | 01801 | 00101 | 00802 | 1, 1 | 1 |
| 6 | GSet | 0 | 01801 | 00101 | 00802 | 01801 | 00101 | 00802 | 1, 1 | 1 |
| 7 | GSet | 0 | 01801 | 00101 | 00802 | 01801 | 00101 | 00802 | 1, 1 | 1 |
| 8 | GSet | 0 | 01801 | 00101 | 00802 | 01801 | 00101 | 00802 | 1, 1 | 1 |
| 9 | GSet | 0 | 01801 | 00101 | 00802 | 01801 | 00101 | 00802 | 1, 1 | 1 |
| 10 | GSet | 0 | 01801 | 00101 | 00802 | 01501 | 00601 | 02301 | 1, 2 | 2 |
| 11 | GSet | 0 | 01801 | 00101 | 00802 | 01501 | 00601 | 02301 | 1, 2 | 2 |
| 12 | GSet | 0 | 01801 | 00101 | 00802 | 01501 | 00601 | 02301 | 1, 2 | 2 |
| 13 | GSet | 0 | 01801 | 00101 | 00802 | 01501 | 00601 | 02301 | 1, 2 | 2 |
| 14 | GSet | 0 | 01801 | 00101 | 00802 | 01501 | 00601 | 02301 | 1, 2 | 2 |
| 15 | GSet | 0 | 01801 | 00101 | 00802 | 01501 | 00601 | 02301 | 1, 2 | 2 |
| 16 | GSet | 0 | 01801 | 00101 | 00802 | 1800103 | 00101 | 00201 | 1, 3 | 3 |
| 17 | GSet | 0 | 01801 | 00101 | 00802 | 1800103 | 00101 | 00201 | 1, 3 | 3 |
| 18 | GSet | 0 | 01801 | 00101 | 00802 | 1800103 | 00101 | 00201 | 1, 3 | 3 |
| 19 | GSet | 0 | 01801 | 00101 | 00802 | 1800103 | 00101 | 00201 | 1, 3 | 3 |
| 20 | GSet | 0 | 01801 | 00101 | 00802 | 1800103 | 00101 | 00201 | 1, 3 | 3 |
| 21 | GSet | 0 | 01801 | 00101 | 00802 | 00101 | 00101 | 00201 | 1, 4 | 4 |
| 22 | GSet | 0 | 01801 | 00101 | 00802 | 00101 | 00101 | 00201 | 1, 4 | 4 |
| 23 | GSet | 0 | 01801 | 00101 | 00802 | 00101 | 00101 | 00201 | 1, 4 | 4 |
| 24 | GSet | 0 | 01801 | 00101 | 00802 | 00101 | 00101 | 00201 | 1, 4 | 4 |
| 25 | GSet | 0 | 01801 | 00101 | 00802 | 00101 | 00101 | 00201 | 1, 4 | 4 |
| 26 | GSet | 0 | 01801 | 00101 | 00802 | 00101 | 00101 | 00201 | 1, 4 | 4 |
| 27 | GSet | 0 | 01801 | 00101 | 00802 | 00101 | 00101 | 00201 | 1, 4 | 4 |
| 28 | GSet | 0 | 01801 | 00101 | 00802 | 02001 | 00401 | 01303 | 1, 5 | 5 |
| 29 | GSet | 0 | 01801 | 00101 | 00802 | 02001 | 00401 | 01303 | 1, 5 | 5 |
| 30 | GSet | 0 | 01801 | 00101 | 00802 | 02001 | 00401 | 01303 | 1, 5 | 5 |
| 31 | GSet | 0 | 01801 | 00101 | 00802 | 02001 | 00401 | 01303 | 1, 5 | 5 |
| 32 | GSet | 0 | 01801 | 00101 | 00802 | 02001 | 00401 | 01303 | 1, 5 | 5 |
| 33 | GSet | 0 | 01801 | 00101 | 00802 | 02001 | 00401 | 01303 | 1, 5 | 5 |
| 34 | GSet | 0 | 01801 | 00101 | 00802 | 02001 | 00401 | 01303 | 1, 5 | 5 |
| 35 | GSet | 0 | 01801 | 00101 | 00802 | 02001 | 00401 | 01303 | 1, 5 | 5 |
| 36 | GSet | 0 | 01801 | 00101 | 00802 | 02001 | 00401 | 01303 | 1, 5 | 5 |
| 37 | GSet | 0 | 01801 | 00101 | 00802 | 02001 | 00401 | 01303 | 1, 5 | 5 |
| 38 | GSet | 0 | 01801 | 00101 | 00802 | 02001 | 00401 | 01303 | 1, 5 | 5 |
| 39 | GSet | 0 | 01801 | 00101 | 00802 | 04901 | 01001 | 01901 | 1, 6 | 6 |
| 40 | GSet | 0 | 01801 | 00101 | 00802 | 04901 | 01001 | 01303 | 1, 6 | 6 |
| 41 | GSet | 0 | 01801 | 00101 | 00802 | 04901 | 01001 | 01901 | 1, 6 | 6 |
| 42 | GSet | 0 | 01801 | 00101 | 00802 | 04901 | 01001 | 01901 | 1, 6 | 6 |
| 43 | GSet | 0 | 01801 | 00101 | 00802 | 00901 | 00101 | 008011 | 1, 7 | 7 |
| 44 | GSet | 0 | 01801 | 00101 | 00802 | 00901 | 00101 | 008011 | 1, 7 | 7 |
| 45 | GSet | 0 | 01801 | 00101 | 00802 | 00901 | 00101 | 008011 | 1, 7 | 7 |
| 46 | GSet | 0 | 01801 | 00101 | 00802 | 00901 | 00101 | 008011 | 1, 7 | 7 |
| 47 | GSet | 0 | 01801 | 00101 | 00802 | 00901 | 00101 | 008011 | 1, 7 | 7 |
| 48 | GSet | 0 | 01801 | 00101 | 00802 | 00901 | 00101 | 008011 | 1, 7 | 7 |
| 49 | GSet | 0 | 01801 | 00101 | 00802 | 00901 | 00101 | 008011 | 1, 7 | 7 |
| 50 | GSet | 0 | 01801 | 00101 | 00802 | 00901 | 00101 | 008011 | 1, 7 | 7 |
| 51 | GSet | 0 | 01801 | 00101 | 00802 | 00901 | 00101 | 008011 | 1, 7 | 7 |
| 52 | GSet | 0 | 01801 | 00101 | 00802 | 00901 | 00101 | 008011 | 1, 7 | 7 |
| 53 | GSet | 0 | 01801 | 00101 | 00802 | 00901 | 00101 | 008011 | 1, 7 | 7 |
| 54 | GSet | 0 | 01801 | 00101 | 00802 | 00601 | 005011 | 00701 | 1, 8 | 8 |
| 55 | GSet | 0 | 01801 | 00101 | 00802 | 00501 | 00301 | 00501 | 1, 9 | 9 |
| 56 | GSet | 0 | 01801 | 00101 | 00802 | 10102 | 00101 | 00201 | 1, 10 | 10 |
| 57 | GSet | 0 | 01801 | 00101 | 00802 | 10102 | 00101 | 00201 | 1, 10 | 10 |
| 58 | GSet | 0 | 01801 | 00101 | 00802 | 10102 | 00101 | 00201 | 1, 10 | 10 |
| 59 | GSet | 0 | 01501 | 00601 | 02301 | 1800103 | 00101 | 00201 | 2, 3 | 12 |
| 60 | GSet | 0 | 01501 | 00601 | 02301 | 1800103 | 00101 | 00201 | 2, 3 | 12 |
| 61 | GSet | 0 | 01501 | 00601 | 02301 | 02001 | 00401 | 01303 | 2, 5 | 13 |
| 62 | GSet | 0 | 01501 | 00601 | 02301 | 02001 | 00401 | 01303 | 2, 5 | 13 |
| 63 | GSet | 0 | 01501 | 00601 | 02301 | 02001 | 00401 | 01303 | 2, 5 | 13 |
| 64 | GSet | 0 | 01501 | 00601 | 02301 | 02001 | 00401 | 01303 | 2, 5 | 13 |
| 65 | GSet | 0 | 01501 | 00601 | 02301 | 04901 | 01001 | 01901 | 2, 6 | 14 |
| 66 | GSet | 0 | 1800103 | 00101 | 00201 | 1800103 | 00101 | 00201 | 3, 3 | 16 |
| 67 | GSet | 0 | 1800103 | 00101 | 00201 | 00101 | 00101 | 00201 | 3, 4 | 17 |
| 68 | GSet | 0 | 1800103 | 00101 | 00201 | 00101 | 00101 | 00201 | 3, 4 | 17 |
| 69 | GSet | 0 | 1800103 | 00101 | 00201 | 02001 | 00401 | 01303 | 3, 5 | 18 |
| 70 | GSet | 0 | 1800103 | 00101 | 00201 | 02001 | 00401 | 01303 | 3, 5 | 18 |
| 71 | GSet | 0 | 1800103 | 00101 | 00201 | 02001 | 00401 | 01303 | 3, 5 | 18 |
| 72 | GSet | 0 | 1800103 | 00101 | 00201 | 04901 | 01001 | 01901 | 3, 6 | 19 |
| 73 | GSet | 0 | 1800103 | 00101 | 00201 | 00901 | 00101 | 008011 | 3, 7 | 20 |
| 74 | GSet | 0 | 00101 | 00101 | 00201 | 02001 | 00401 | 01303 | 4, 5 | 22 |
| 75 | GSet | 0 | 00101 | 00101 | 00201 | 02001 | 00401 | 01303 | 4, 5 | 22 |
| 76 | GSet | 0 | 00101 | 00101 | 00201 | 02001 | 00401 | 01303 | 4, 5 | 22 |
| 77 | GSet | 0 | 00101 | 00101 | 00201 | 02001 | 00401 | 01303 | 4, 5 | 22 |
| 78 | GSet | 0 | 00101 | 00101 | 00201 | 02001 | 00401 | 01303 | 4, 5 | 22 |
| 79 | GSet | 0 | 00101 | 00101 | 00201 | 02001 | 00401 | 01303 | 4, 5 | 22 |
| 80 | GSet | 0 | 00101 | 00101 | 00201 | 04901 | 01001 | 01901 | 4, 6 | 23 |
| 81 | GSet | 0 | 00101 | 00101 | 00201 | 04901 | 01001 | 01901 | 4, 6 | 23 |
| 82 | GSet | 0 | 00101 | 00101 | 00201 | 04901 | 01001 | 01901 | 4, 6 | 23 |
| 83 | GSet | 0 | 00101 | 00101 | 00201 | 00901 | 00101 | 008011 | 4, 7 | 24 |
| 84 | GSet | 0 | 00101 | 00101 | 00201 | 00901 | 00101 | 008011 | 4, 7 | 24 |
| 85 | GSet | 0 | 02001 | 00401 | 01303 | 02001 | 00401 | 01303 | 5, 5 | 26 |
| 86 | GSet | 0 | 02001 | 00401 | 01303 | 02001 | 00401 | 01303 | 5, 5 | 26 |
| 87 | GSet | 0 | 02001 | 00401 | 01303 | 02001 | 00401 | 01303 | 5, 5 | 26 |
| 88 | GSet | 0 | 02001 | 00401 | 01303 | 04901 | 01001 | 01303 | 5, 6 | 27 |
| 89 | GSet | 0 | 02001 | 00401 | 01303 | 00901 | 00101 | 008011 | 5, 7 | 28 |
| 90 | GSet | 0 | 02001 | 00401 | 01303 | 10102 | 00101 | 00201 | 5, 10 | 29 |
| 91 | GSet | 0 | 04901 | 01001 | 01901 | 04901 | 01001 | 01901 | 6, 6 | 30 |
| 92 | GSet | 0 | 04901 | 01001 | 01901 | 04901 | 01001 | 01901 | 6, 6 | 30 |
| 93 | GSet | 0 | 04901 | 01001 | 01901 | 04901 | 01001 | 01901 | 6, 6 | 30 |
| 94 | GSet | 0 | 04901 | 01001 | 01901 | 00901 | 00101 | 008011 | 6, 7 | 31 |
| 95 | GSet | 0 | 04901 | 01001 | 01901 | 00501 | 00301 | 00501 | 6, 9 | 32 |
| 96 | GSet | 0 | 04901 | 01001 | 01901 | 00501 | 00301 | 00501 | 6, 9 | 32 |
| 97 | GSet | 0 | 00901 | 00101 | 008011 | 00901 | 00101 | 008011 | 7, 7 | 34 |
| 98 | GSet | 0 | 00901 | 00101 | 008011 | 00901 | 00101 | 008011 | 7, 7 | 34 |
| 99 | GSet | 1 | 01801 | 00101 | 00802 | 01801 | 00101 | 00802 | 1, 1 | 1 |
| 100 | GSet | 1 | 01801 | 00101 | 00802 | 01801 | 00101 | 00802 | 1, 1 | 1 |
| 101 | GSet | 1 | 01801 | 00101 | 00802 | 01801 | 00101 | 00802 | 1, 1 | 1 |
| 102 | GSet | 1 | 01801 | 00101 | 00802 | 01801 | 00101 | 00802 | 1, 1 | 1 |
| 103 | GSet | 1 | 01801 | 00101 | 00802 | 01801 | 00101 | 00802 | 1, 1 | 1 |
| 104 | GSet | 1 | 01801 | 00101 | 00802 | 01801 | 00101 | 00802 | 1, 1 | 1 |
| 105 | GSet | 1 | 01801 | 00101 | 00802 | 01801 | 00101 | 00802 | 1, 1 | 1 |
| 106 | GSet | 1 | 01801 | 00101 | 00802 | 01801 | 00101 | 00802 | 1, 1 | 1 |
| 107 | GSet | 1 | 01801 | 00101 | 00802 | 01801 | 00101 | 00802 | 1, 1 | 1 |
| 108 | GSet | 1 | 01801 | 00101 | 00802 | 01801 | 00101 | 00802 | 1, 1 | 1 |
| 109 | GSet | 1 | 01801 | 00101 | 00802 | 01801 | 00101 | 00802 | 1, 1 | 1 |
| 110 | GSet | 1 | 01801 | 00101 | 00802 | 01801 | 00101 | 00802 | 1, 1 | 1 |
| 111 | GSet | 1 | 01801 | 00101 | 00802 | 01801 | 00101 | 00802 | 1, 1 | 1 |
| 112 | GSet | 1 | 01801 | 00101 | 00802 | 01801 | 00101 | 00802 | 1, 1 | 1 |
| 113 | GSet | 1 | 01801 | 00101 | 00802 | 01801 | 00101 | 00802 | 1, 1 | 1 |
| 114 | GSet | 1 | 01801 | 00101 | 00802 | 01801 | 00101 | 00802 | 1, 1 | 1 |
| 115 | GSet | 1 | 01801 | 00101 | 00802 | 01801 | 00101 | 00802 | 1, 1 | 1 |
| 116 | GSet | 1 | 01801 | 00101 | 00802 | 01801 | 00101 | 00802 | 1, 1 | 1 |
| 117 | GSet | 1 | 01801 | 00101 | 00802 | 01801 | 00101 | 00802 | 1, 1 | 1 |
| 118 | GSet | 1 | 01801 | 00101 | 00802 | 01801 | 00101 | 00802 | 1, 1 | 1 |
| 119 | GSet | 1 | 01801 | 00101 | 00802 | 01801 | 00101 | 00802 | 1, 1 | 1 |
| 120 | GSet | 1 | 01801 | 00101 | 00802 | 01801 | 00101 | 00802 | 1, 1 | 1 |
| 121 | GSet | 1 | 01801 | 00101 | 00802 | 01801 | 00101 | 00802 | 1, 1 | 1 |
| 122 | GSet | 1 | 01801 | 00101 | 00802 | 01801 | 00101 | 00802 | 1, 1 | 1 |
| 123 | GSet | 1 | 01801 | 00101 | 00802 | 01801 | 00101 | 00802 | 1, 1 | 1 |
| 124 | GSet | 1 | 01801 | 00101 | 00802 | 01801 | 00101 | 00802 | 1, 1 | 1 |
| 125 | GSet | 1 | 01801 | 00101 | 00802 | 01801 | 00101 | 00802 | 1, 1 | 1 |
| 126 | GSet | 1 | 01801 | 00101 | 00802 | 01801 | 00101 | 00802 | 1, 1 | 1 |
| 127 | GSet | 1 | 01801 | 00101 | 00802 | 01501 | 00601 | 02301 | 1, 2 | 2 |
| 128 | GSet | 1 | 01801 | 00101 | 00802 | 01501 | 00601 | 02301 | 1, 2 | 2 |
| 129 | GSet | 1 | 01801 | 00101 | 00802 | 1800103 | 00101 | 00201 | 1, 3 | 3 |
| 130 | GSet | 1 | 01801 | 00101 | 00802 | 1800103 | 00101 | 00201 | 1, 3 | 3 |
| 131 | GSet | 1 | 01801 | 00101 | 00802 | 1800103 | 00101 | 00201 | 1, 3 | 3 |
| 132 | GSet | 1 | 01801 | 00101 | 00802 | 1800103 | 00101 | 00201 | 1, 3 | 3 |
| 133 | GSet | 1 | 01801 | 00101 | 00802 | 1800103 | 00101 | 00201 | 1, 3 | 3 |
| 134 | GSet | 1 | 01801 | 00101 | 00802 | 1800103 | 00101 | 00201 | 1, 3 | 3 |
| 135 | GSet | 1 | 01801 | 00101 | 00802 | 1800103 | 00101 | 00201 | 1, 3 | 3 |
| 136 | GSet | 1 | 01801 | 00101 | 00802 | 1800103 | 00101 | 00201 | 1, 3 | 3 |
| 137 | GSet | 1 | 01801 | 00101 | 00802 | 1800103 | 00101 | 00201 | 1, 3 | 3 |
| 138 | GSet | 1 | 01801 | 00101 | 00802 | 1800103 | 00101 | 00201 | 1, 3 | 3 |
| 139 | GSet | 1 | 01801 | 00101 | 00802 | 1800103 | 00101 | 00201 | 1, 3 | 3 |
| 140 | GSet | 1 | 01801 | 00101 | 00802 | 1800103 | 00101 | 00201 | 1, 3 | 3 |
| 141 | GSet | 1 | 01801 | 00101 | 00802 | 1800103 | 00101 | 00201 | 1, 3 | 3 |
| 142 | GSet | 1 | 01801 | 00101 | 00802 | 1800103 | 00101 | 00201 | 1, 3 | 3 |
| 143 | GSet | 1 | 01801 | 00101 | 00802 | 00101 | 00101 | 00201 | 1, 4 | 4 |
| 144 | GSet | 1 | 01801 | 00101 | 00802 | 00101 | 00101 | 00201 | 1, 4 | 4 |
| 145 | GSet | 1 | 01801 | 00101 | 00802 | 00101 | 00101 | 00201 | 1, 4 | 4 |
| 146 | GSet | 1 | 01801 | 00101 | 00802 | 00101 | 00101 | 00201 | 1, 4 | 4 |
| 147 | GSet | 1 | 01801 | 00101 | 00802 | 00101 | 00101 | 00201 | 1, 4 | 4 |
| 148 | GSet | 1 | 01801 | 00101 | 00802 | 00101 | 00101 | 00201 | 1, 4 | 4 |
| 149 | GSet | 1 | 01801 | 00101 | 00802 | 00101 | 00101 | 00201 | 1, 4 | 4 |
| 150 | GSet | 1 | 01801 | 00101 | 00802 | 00101 | 00101 | 00201 | 1, 4 | 4 |
| 151 | GSet | 1 | 01801 | 00101 | 00802 | 00101 | 00101 | 00201 | 1, 4 | 4 |
| 152 | GSet | 1 | 01801 | 00101 | 00802 | 00101 | 00101 | 00201 | 1, 4 | 4 |
| 153 | GSet | 1 | 01801 | 00101 | 00802 | 00101 | 00101 | 00201 | 1, 4 | 4 |
| 154 | GSet | 1 | 01801 | 00101 | 00802 | 00101 | 00101 | 00201 | 1, 4 | 4 |
| 155 | GSet | 1 | 01801 | 00101 | 00802 | 04901 | 01001 | 01901 | 1, 6 | 6 |
| 156 | GSet | 1 | 01801 | 00101 | 00802 | 04901 | 01001 | 01901 | 1, 6 | 6 |
| 157 | GSet | 1 | 01801 | 00101 | 00802 | 04901 | 01001 | 01901 | 1, 6 | 6 |
| 158 | GSet | 1 | 01801 | 00101 | 00802 | 04901 | 01001 | 01901 | 1, 6 | 6 |
| 159 | GSet | 1 | 01801 | 00101 | 00802 | 04901 | 01001 | 01901 | 1, 6 | 6 |
| 160 | GSet | 1 | 01801 | 00101 | 00802 | 04901 | 01001 | 01901 | 1, 6 | 6 |
| 161 | GSet | 1 | 01801 | 00101 | 00802 | 00901 | 00101 | 008011 | 1, 7 | 7 |
| 162 | GSet | 1 | 01801 | 00101 | 00802 | 00901 | 00101 | 008011 | 1, 7 | 7 |
| 163 | GSet | 1 | 01801 | 00101 | 00802 | 00501 | 00301 | 00501 | 1, 9 | 9 |
| 164 | GSet | 1 | 01801 | 00101 | 00802 | 10102 | 00101 | 00201 | 1, 10 | 10 |
| 165 | GSet | 1 | 01801 | 00101 | 00802 | 10102 | 00101 | 00201 | 1, 10 | 10 |
| 166 | GSet | 1 | 01801 | 00101 | 00802 | 10102 | 00101 | 00201 | 1, 10 | 10 |
| 167 | GSet | 1 | 01801 | 00101 | 00802 | 10102 | 00101 | 00201 | 1, 10 | 10 |
| 168 | GSet | 1 | 01801 | 00101 | 00802 | 10102 | 00101 | 00201 | 1, 10 | 10 |
| 169 | GSet | 1 | 01801 | 00101 | 00802 | 10102 | 00101 | 00201 | 1, 10 | 10 |
| 170 | GSet | 1 | 01801 | 00101 | 00802 | 10102 | 00101 | 00201 | 1, 10 | 10 |
| 171 | GSet | 1 | 01801 | 00101 | 00802 | 10102 | 00101 | 00201 | 1, 10 | 10 |
| 172 | GSet | 1 | 01801 | 00101 | 00802 | 10102 | 00101 | 00201 | 1, 10 | 10 |
| 173 | GSet | 1 | 01801 | 00101 | 00802 | 10102 | 00101 | 00201 | 1, 10 | 10 |
| 174 | GSet | 1 | 01501 | 00601 | 02301 | 01501 | 00601 | 02301 | 2, 2 | 11 |
| 175 | GSet | 1 | 01501 | 00601 | 02301 | 00901 | 00101 | 008011 | 2, 7 | 15 |
| 176 | GSet | 1 | 1800103 | 00101 | 00201 | 00101 | 00101 | 00201 | 3, 4 | 17 |
| 177 | GSet | 1 | 1800103 | 00101 | 00201 | 04901 | 01001 | 01901 | 3, 6 | 19 |
| 178 | GSet | 1 | 00101 | 00101 | 00201 | 00101 | 00101 | 00201 | 4, 4 | 21 |
| 179 | GSet | 1 | 00101 | 00101 | 00201 | 00101 | 00101 | 00201 | 4, 4 | 21 |
| 180 | GSet | 1 | 00101 | 00101 | 00201 | 00101 | 00101 | 00201 | 4, 4 | 21 |
| 181 | GSet | 1 | 00101 | 00101 | 00201 | 00101 | 00101 | 00201 | 4, 4 | 21 |
| 182 | GSet | 1 | 00101 | 00101 | 00201 | 02001 | 00401 | 01303 | 4, 5 | 22 |
| 183 | GSet | 1 | 00101 | 00101 | 00201 | 04901 | 01001 | 01901 | 4, 6 | 23 |
| 184 | GSet | 1 | 00101 | 00101 | 00201 | 04901 | 01001 | 01901 | 4, 6 | 23 |
| 185 | GSet | 1 | 00101 | 00101 | 00201 | 04901 | 01001 | 01901 | 4, 6 | 23 |
| 186 | GSet | 1 | 00101 | 00101 | 00201 | 04901 | 01001 | 01901 | 4, 6 | 23 |
| 187 | GSet | 1 | 00101 | 00101 | 00201 | 04901 | 01001 | 01901 | 4, 6 | 23 |
| 188 | GSet | 1 | 01801 | 00101 | 00201 | 04901 | 01001 | 01901 | 4, 6 | 23 |
| 189 | GSet | 1 | 00101 | 00101 | 00201 | 04901 | 01001 | 01901 | 4, 6 | 23 |
| 190 | GSet | 1 | 00101 | 00101 | 00201 | 10102 | 00101 | 00201 | 4, 10 | 25 |
| 191 | GSet | 1 | 04901 | 01001 | 01901 | 04901 | 01001 | 01901 | 6, 6 | 30 |
| 192 | GSet | 1 | 04901 | 01001 | 01901 | 04901 | 01001 | 01901 | 6, 6 | 30 |
| 193 | GSet | 1 | 04901 | 01001 | 01901 | 04901 | 01001 | 01901 | 6, 6 | 30 |
| 194 | GSet | 1 | 04901 | 01001 | 01901 | 04901 | 01001 | 01901 | 6, 6 | 30 |
| 195 | GSet | 1 | 04901 | 01001 | 01901 | 04901 | 01001 | 01901 | 6, 6 | 30 |
| 196 | GSet | 1 | 04901 | 01001 | 01901 | 10102 | 00101 | 00201 | 6, 10 | 33 |
| 1 | BC | 0 | 01801 | 00101 | 00802 | 01801 | 00101 | 00201 | 1, 2 | 1 |
| 2 | BC | 0 | 01801 | 00101 | 00802 | 01801 | 00101 | 00201 | 1, 2 | 1 |
| 5 | BC | 0 | 01801 | 00101 | 00201 | 01801 | 00101 | 00201 | 2, 2 | 2 |
| 3 | BC | 0 | 01501 | 00601 | 00301 | 01501 | 00601 | 02301 | 3, 5 | 3 |
| 4 | BC | 0 | 00201 | 00901 | 00101 | 01501 | 00601 | 02301 | 4, 5 | 4 |
| 6 | BC | 1 | 01801 | 00101 | 00802 | 01801 | 00101 | 00201 | 1, 2 | 1 |
| 7 | BC | 1 | 01801 | 00101 | 00802 | 01801 | 00101 | 00201 | 1, 2 | 1 |
| 8 | BC | 1 | 01801 | 00101 | 00802 | 01801 | 00101 | 00201 | 1, 2 | 1 |
| 9 | BC | 1 | 01801 | 00101 | 00802 | 01801 | 00101 | 00201 | 1, 2 | 1 |
| 10 | BC | 1 | 01801 | 00101 | 00201 | 01801 | 00101 | 00201 | 2, 2 | 2 |
| 1 | GSch | 0 | 00101 | 00101 | 00201 | 00101 | 00101 | 00201 | 1, 1 | 1 |
| 2 | GSch | 0 | 00101 | 00101 | 00201 | 00101 | 00101 | 00201 | 1, 1 | 1 |
| 3 | GSch | 0 | 00101 | 00101 | 00201 | 01201 | 00101 | 00201 | 1, 2 | 2 |
| 4 | GSch | 0 | 00101 | 00101 | 00201 | 01201 | 00101 | 00201 | 1, 2 | 2 |
| 5 | GSch | 0 | 00101 | 00101 | 00201 | 01201 | 00101 | 00201 | 1, 2 | 2 |
| 6 | GSch | 0 | 00101 | 00101 | 00201 | 00601 | 00401 | 01303 | 1, 3 | 3 |
| 7 | GSch | 0 | 00101 | 00101 | 00201 | 01301 | 00301 | 00501 | 1, 5 | 5 |
| 8 | GSch | 0 | 01201 | 00101 | 00201 | 01201 | 00101 | 00201 | 2, 2 | 8 |
| 9 | GSch | 0 | 01201 | 00101 | 00201 | 02301 | 00301 | 00501 | 2, 6 | 9 |
| 10 | GSch | 0 | 00601 | 00401 | 01303 | 01301 | 00101 | 00201 | 3, 4 | 12 |
| 11 | GSch | 0 | 00601 | 00401 | 01303 | 01301 | 00101 | 00201 | 3, 4 | 12 |
| 12 | GSch | 0 | 00601 | 00401 | 01303 | 01301 | 00101 | 00201 | 3, 4 | 12 |
| 13 | GSch | 0 | 00601 | 00401 | 01303 | 01301 | 00101 | 00201 | 3, 4 | 12 |
| 14 | GSch | 0 | 00601 | 00401 | 01303 | 01301 | 00101 | 00201 | 3, 4 | 12 |
| 15 | GSch | 0 | 00601 | 00401 | 01303 | 01301 | 00301 | 00501 | 3, 5 | 13 |
| 16 | GSch | 0 | 00601 | 00401 | 01303 | 01301 | 00301 | 00501 | 3, 5 | 13 |
| 17 | GSch | 0 | 00601 | 00401 | 01303 | 01301 | 00301 | 00501 | 3, 5 | 13 |
| 18 | GSch | 0 | 00601 | 00401 | 01303 | 01301 | 00301 | 00501 | 3, 5 | 13 |
| 19 | GSch | 0 | 00601 | 00401 | 01303 | 01301 | 00301 | 00501 | 3, 5 | 13 |
| 20 | GSch | 0 | 00601 | 00401 | 01303 | 00901 | 00101 | 08011 | 3, 7 | 15 |
| 21 | GSch | 0 | 01301 | 00101 | 00201 | 01301 | 00101 | 00201 | 4, 4 | 19 |
| 22 | GSch | 0 | 01301 | 00301 | 00501 | 01201 | 00101 | 00201 | 5, 2 | 21 |
| 23 | GSch | 0 | 01301 | 00301 | 00501 | 01301 | 00101 | 00201 | 5, 4 | 22 |
| 24 | GSch | 0 | 01301 | 00301 | 00501 | 01301 | 00101 | 00201 | 5, 4 | 22 |
| 25 | GSch | 0 | 01301 | 00301 | 00501 | 01301 | 00301 | 00501 | 5, 5 | 23 |
| 26 | GSch | 0 | 01301 | 00301 | 00501 | 01301 | 00301 | 00501 | 5, 5 | 23 |
| 27 | GSch | 0 | 01301 | 00301 | 00501 | 01501 | 00601 | 02201 | 5, 8 | 25 |
| 28 | GSch | 0 | 01301 | 00301 | 00501 | 01501 | 00601 | 00301 | 5, 10 | 26 |
| 29 | GSch | 0 | 02301 | 00301 | 00501 | 02301 | 00301 | 00501 | 6,6 | 28 |
| 30 | GSch | 0 | 901 | 00101 | 08011 | 00901 | 00101 | 08011 | 7, 7 | 29 |
| 31 | GSch | 1 | 00101 | 00101 | 00201 | 00101 | 00101 | 00201 | 1, 1 | 1 |
| 32 | GSch | 1 | 00101 | 00101 | 00201 | 00101 | 00101 | 00201 | 1, 1 | 1 |
| 33 | GSch | ÅK | 00101 | 00101 | 00201 | 00101 | 00101 | 00201 | 1, 1 | 1 |
| 34 | GSch | ÅK | 00101 | 00101 | 00201 | 00101 | 00101 | 00201 | 1, 1 | 1 |
| 35 | GSch | E | 00101 | 00101 | 00201 | 00101 | 00101 | 00201 | 1, 1 | 1 |
| 36 | GSch | EK | 00101 | 00101 | 00201 | 00101 | 00101 | 00201 | 1, 1 | 1 |
| 37 | GSch | UCP | 00101 | 00101 | 00201 | 00101 | 00101 | 00201 | 1, 1 | 1 |
| 38 | GSch | UCP | 00101 | 00101 | 00201 | 00101 | 00101 | 00201 | 1, 1 | 1 |
| 39 | GSch | UCP | 00101 | 00101 | 00201 | 00101 | 00101 | 00201 | 1, 1 | 1 |
| 40 | GSch | 1 | 00101 | 00101 | 00201 | 01201 | 00101 | 00201 | 1, 2 | 2 |
| 41 | GSch | ÅK | 00101 | 00101 | 00201 | 01201 | 00101 | 00201 | 1, 2 | 2 |
| 42 | GSch | EK | 00101 | 00101 | 00201 | 01201 | 00101 | 00201 | 1, 2 | 2 |
| 43 | GSch | EK | 00101 | 00101 | 00201 | 01201 | 00101 | 00201 | 1, 2 | 2 |
| 44 | GSch | PEC | 00101 | 00101 | 00201 | 01201 | 00101 | 00201 | 1, 2 | 2 |
| 45 | GSch | EK | 00101 | 00101 | 00201 | 00601 | 00401 | 01303 | 1, 3 | 3 |
| 46 | GSch | EK | 00101 | 00101 | 00201 | 00601 | 00401 | 01303 | 1, 3 | 3 |
| 47 | GSch | EK | 00101 | 00101 | 00201 | 00601 | 00401 | 01303 | 1, 3 | 3 |
| 48 | GSch | EK | 00101 | 00101 | 00201 | 00601 | 00401 | 01303 | 1, 3 | 3 |
| 49 | GSch | EK | 00101 | 00101 | 00201 | 00601 | 00401 | 01303 | 1, 3 | 3 |
| 50 | GSch | PEC | 00101 | 00101 | 00201 | 00601 | 00401 | 01303 | 1, 3 | 3 |
| 51 | GSch | UCP | 00101 | 00101 | 00201 | 00601 | 00401 | 01303 | 1, 3 | 3 |
| 52 | GSch | 1 | 00101 | 00101 | 00201 | 01301 | 00101 | 00201 | 1, 4 | 4 |
| 53 | GSch | EK | 00101 | 00101 | 00201 | 01301 | 00101 | 00201 | 1, 4 | 4 |
| 54 | GSch | PEC | 00101 | 00101 | 00201 | 01301 | 00101 | 00201 | 1, 4 | 4 |
| 55 | GSch | 1 | 00101 | 00101 | 00201 | 01301 | 00301 | 00501 | 1, 5 | 5 |
| 56 | GSch | ÅK | 00101 | 00101 | 00201 | 01301 | 00301 | 00501 | 1, 5 | 5 |
| 57 | GSch | EK | 00101 | 00101 | 00201 | 01301 | 00301 | 00501 | 1, 5 | 5 |
| 58 | GSch | EK | 00101 | 00101 | 00201 | 01301 | 00301 | 00501 | 1, 5 | 5 |
| 59 | GSch | UCP | 00101 | 00101 | 00201 | 01301 | 00301 | 00501 | 1, 5 | 5 |
| 60 | GSch | ÅK | 00101 | 00101 | 00201 | 02301 | 00301 | 00501 | 1, 6 | 6 |
| 61 | GSch | EK | 00101 | 00101 | 00201 | 02301 | 00301 | 00501 | 1, 6 | 6 |
| 62 | GSch | EK | 00101 | 00101 | 00201 | 02301 | 00301 | 00501 | 1,6 | 6 |
| 63 | GSch | PEC | 00101 | 00101 | 00201 | 02301 | 00301 | 00501 | 1, 6 | 6 |
| 64 | GSch | PEC | 00101 | 00101 | 00201 | 01501 | 00601 | 00301 | 1, 10 | 7 |
| 65 | GSch | ÅI | 01201 | 00101 | 00201 | 01201 | 00101 | 00201 | 2, 2 | 8 |
| 66 | GSch | ÅI | 01201 | 00101 | 00201 | 01201 | 00101 | 00201 | 2, 2 | 8 |
| 67 | GSch | EK | 01201 | 00101 | 00201 | 01201 | 00101 | 00201 | 2, 2 | 8 |
| 68 | GSch | ÅK | 01201 | 00101 | 00201 | 02301 | 00301 | 00501 | 2, 6 | 9 |
| 69 | GSch | ÅK | 01201 | 00101 | 00201 | 02301 | 00301 | 00501 | 2, 6 | 9 |
| 70 | GSch | ÅK | 01201 | 00101 | 00201 | 02301 | 00301 | 00501 | 2, 6 | 9 |
| 71 | GSch | E | 01201 | 00101 | 00201 | 02301 | 00301 | 00501 | 2, 6 | 9 |
| 72 | GSch | PEC | 01201 | 00101 | 00201 | 02301 | 00301 | 00501 | 2, 6 | 9 |
| 73 | GSch | ÅK | 00601 | 00401 | 01303 | 01201 | 00101 | 00201 | 3, 2 | 10 |
| 74 | GSch | PEC | 00601 | 00401 | 01303 | 01201 | 00101 | 00201 | 3, 2 | 10 |
| 75 | GSch | 1 | 00601 | 00401 | 01303 | 00601 | 00401 | 01303 | 3, 3 | 11 |
| 76 | GSch | EK | 00601 | 00401 | 01303 | 00601 | 00401 | 01303 | 3, 3 | 11 |
| 77 | GSch | PEC | 00601 | 00401 | 01303 | 00601 | 00401 | 01303 | 3, 3 | 11 |
| 78 | GSch | PEC | 00601 | 00401 | 01303 | 00601 | 00401 | 01303 | 3, 3 | 11 |
| 79 | GSch | PEC | 00601 | 00401 | 01303 | 00601 | 00401 | 01303 | 3, 3 | 11 |
| 80 | GSch | UCP | 00601 | 00401 | 01303 | 00601 | 00401 | 01303 | 3, 3 | 11 |
| 81 | GSch | ÅK | 00601 | 00401 | 01303 | 01301 | 00301 | 00501 | 3, 5 | 13 |
| 82 | GSch | ÅK | 00601 | 00401 | 01303 | 01301 | 00301 | 00501 | 3, 5 | 13 |
| 83 | GSch | EK | 00601 | 00401 | 01303 | 01301 | 00301 | 00501 | 3, 5 | 13 |
| 84 | GSch | EK | 00601 | 00401 | 01303 | 01301 | 00301 | 00501 | 3, 5 | 13 |
| 85 | GSch | UCP | 00601 | 00401 | 01303 | 01301 | 00301 | 00501 | 3, 5 | 13 |
| 86 | GSch | UCP | 00601 | 00401 | 01303 | 01301 | 00301 | 00501 | 3, 5 | 13 |
| 87 | GSch | ÅK | 00601 | 00401 | 01303 | 02301 | 00301 | 00501 | 3, 6 | 14 |
| 88 | GSch | PEC | 00601 | 00401 | 01303 | 02301 | 00301 | 00501 | 3, 6 | 14 |
| 89 | GSch | UCP | 00601 | 00401 | 01303 | 02301 | 00301 | 00501 | 3, 6 | 14 |
| 90 | GSch | UCP | 00601 | 00401 | 01303 | 02301 | 00301 | 00501 | 3, 6 | 14 |
| 91 | GSch | E | 00601 | 00401 | 01303 | 901 | 101 | 8011 | 3, 7 | 15 |
| 92 | GSch | ÅK | 00601 | 00401 | 01303 | 01501 | 00601 | 02201 | 3, 8 | 16 |
| 93 | GSch | ÅK | 00601 | 00401 | 01303 | 2001 | 401 | 1303 | 3, 9 | 17 |
| 94 | GSch | ÅI | 01301 | 00101 | 00201 | 01201 | 00101 | 00201 | 4, 2 | 18 |
| 95 | GSch | ÅK | 01301 | 00101 | 00201 | 01301 | 00101 | 00201 | 4, 4 | 19 |
| 96 | GSch | PEC | 01301 | 00101 | 00201 | 02301 | 00301 | 00501 | 4, 6 | 20 |
| 97 | GSch | UCP | 01301 | 00101 | 00201 | 02301 | 00301 | 00501 | 4, 6 | 20 |
| 98 | GSch | ÅK | 01301 | 00301 | 00501 | 01201 | 00101 | 00201 | 5, 2 | 21 |
| 99 | GSch | EK | 01301 | 00301 | 00501 | 01201 | 00101 | 00201 | 5, 2 | 21 |
| 100 | GSch | EK | 01301 | 00301 | 00501 | 01201 | 00101 | 00201 | 5, 2 | 21 |
| 101 | GSch | UCP | 01301 | 00301 | 00501 | 01201 | 00101 | 00201 | 5, 2 | 21 |
| 102 | GSch | UCP | 01301 | 00301 | 00501 | 02301 | 00301 | 00501 | 5, 6 | 24 |
| 103 | GSch | UCP | 01301 | 00301 | 00501 | 02301 | 00301 | 00501 | 5, 6 | 24 |
| 104 | GSch | 1 | 01301 | 00301 | 00501 | 01501 | 00601 | 02201 | 5, 8 | 25 |
| 105 | GSch | ÅK | 01301 | 00301 | 00501 | 01501 | 00601 | 02201 | 5, 8 | 25 |
| 106 | GSch | ÅK | 01301 | 00301 | 00501 | 01501 | 00601 | 02201 | 5, 8 | 25 |
| 107 | GSch | ÅK | 01301 | 00301 | 00501 | 01501 | 00601 | 02201 | 5, 8 | 25 |
| 108 | GSch | ÅK | 02301 | 00301 | 00501 | 01501 | 00601 | 02201 | 6, 8 | 27 |
| 109 | GSch | E | 01501 | 00601 | 00301 | 01501 | 00601 | 02201 | 10, 8 | 30 |
| 110 | GSch | EK | 01501 | 00601 | 00301 | 01501 | 00601 | 02201 | 10, 8 | 31 |
