# Supplementary material for: DLA Class II Alleles Are Associated with Risk for Canine Symmetrical Lupoid Onychodystropy (SLO)
Source: PLoS One. 2010 Aug 23;5(8):e12332. doi: 10.1371/journal.pone.0012332 (PMC2925901; doi:10.1371/journal.pone.0012332)
Supplement: Table S7 — The inclusion criteria used for giant schnauzers. (0.03 MB DOC) [file pone.0012332.s007.doc]

| Code | Description |
| --- | --- |
| E | Claw fracture one claw one time |
| EK | Claw fracture less or no more than two times, with deviant claw quality, eventually secondary caused claw infections |
| ÅK | Returning claw fractures three times or more, with deviant claw quality, eventually secondary caused claw infections |
| ÅI | Returning primary claw infections on many claws, with no visible claw injury |
| SLO | Symmetrical lupoid onychodystrophy |
| PEC | Squamous cell carcinoma or other tumor |
| UCP | Unclassified claw problems |
